# Early childhood and early adolescent predictors of internalising symptoms in adolescents: findings from a longitudinal study in a high-risk South African environment

**DOI:** 10.1007/s00127-026-03048-w

**Published:** 2026-02-11

**Authors:** Stefani Du Toit, Katharina Haag, Mark Tomlinson, Lorraine Sherr, Marguerite Marlow, Jackie Stewart, Sarah Skeen

**Affiliations:** 1 Institute for Life Course Health Research, Department of Global Health, Stellenbosch University, Cape Town, South Africa; 2 Institute for Global Health, University College London, London, UK; 3 School of Nursing and Midwifery, Queens University, Belfast, UK; 4 The Division of Global Surgery, Department of Surgery, University of Cape Town, Cape Town, South Africa; 5 Department of Social Work and Social Policy, Faculty of Humanities, Strathclyde University, Glasgow, Scotland; 6 Mental Health, Alcohol, SubstanceUse and Tobacco Research Unit (MASTRU), South African Medical Research Council, Cape Town, South Africa

**Keywords:** Adolescents; internalising symptoms, High-Risk content, Longitudinal study

## Abstract

**Purpose:**

This study investigates predictors of internalising symptoms among adolescents aged 16 to 19 years in a high-risk context in South Africa. Specifically, it explores early childhood (antenatal to 18 months postpartum), and early adolescent (13 to 14 years) predictors of internalising symptoms measured during later adolescence (16–19 years), aiming to identify key factors influencing mental health outcomes in this vulnerable population.

**Methods:**

Utilising a unique 18-year longitudinal dataset, we included a total of 314 adolescent participants from South Africa in the analysis and employed an adaptive elastic net regularised regression to analyse the effects of 18 predictors from early childhood and early adolescence on internalising symptoms at ages 16 to 19 years. The broadband scale for “internalising” from the Youth Self Report (ages 11–18) was used as the outcome measure. Data collected at five time points across three phases of the longitudinal study were included in the analysis.

**Results:**

Key predictors of internalising symptoms were female sex (β=-4.30; 95% CI [-4.42;4.19]). Early childhood predictors with significant associations were maternal depression (β = 1.70; 95% CI [1.56;1.84]) and caregiver employment (β=-0.37; 95% CI [-0.46;-0.29]). In early adolescence, significant predictors included informal house type (β = 0.82; 95% CI [0.71;0.93]), caregiver alcohol use (β = 0.74; 95% CI [0.67;0.81]), exposure to violence (β = 0.73; 95% CI [0.67;0.78]), friend support (β=-0.61; 95% CI [-0.67;-0.55]), food insecurity (β = 0.51; 95% CI [0.46;0.56]), family support (β=-0.33; 95% CI [-0.37;-0.29]), and self-esteem (β=-0.33; 95% CI [-0.37;-0.29]).

**Conclusion:**

This study identifies key predictors of internalising symptoms in adolescents from high-risk context, focusing on caregiver variables and social connections. Maternal / Primary cargiver depression and caregiver unemployment in early childhood have lasting effects, highlighting the need for early intervention. In early adolescence, factors such as social environment and caregiver stability are crucial. These insights can inform targeted interventions and policies to support adolescent mental health in high-risk contexts.

**Supplementary Information:**

The online version contains supplementary material available at 10.1007/s00127-026-03048-w.

## Background

Adolescence is a crucial developmental period characterised by significant physical, cognitive, and emotional changes [1], and a time where mental health conditions often emerge [2]. Poverty has been suggested to be a critical determinant of poor mental health outcomes among the adolescent population [3–9]. In high-adversity contexts, adolescents face multiple associated secondary challenges (e.g., exposure to community violence, poor family functioning) and often have limited access to resources that could help them cope effectively (e.g., mental health services) [10–12].

A critical lack of evidence on prevalence rates of mental health conditions among adolescents in low- and middle-income countries (LMIC) exists, especially in sub-Saharan African (SSA) countries, and particularly for adolescents living in adverse contexts such as informal settlements, where data coverage is almost non-existent [13]. Despite the lack of availability of epidemiological data on internalising symptoms in SSA, it is estimated that between 15% and 41% of adolescents in South Africa experiences symptoms of mental health conditions [14–16].

This study focuses on adolescents in South Africa as it is a country with high rates of poverty, unemployment, and community violence, which are key risk factors for internalising symptoms in adolescents. The prevalence of mental health symptoms in this population is high, but there is a lack of research on the specific predictors of poor mental health in these high-risk settings.

Generally, a range of risk factors have been established for adolescent internalising symptoms [17–22]. However, high poverty, a risk factor almost universally present in adverse LMIC contexts, has been suggested to influence the mental health of adolescents directly, as well as through various indirect pathways that are likely not captured by HIC studies. As a result, adolescents living in poverty in LMICs often experience challenging conditions directly linked to such conditions, such as food insecurity or lack of stable housing [23]. Also, they may also face secondary consequences, including chronic stress, poor family functioning, family instability, and/or parental substance use and other mental health conditions [24–26]. Furthermore, they are faced with broader poverty-associated challenges outside the home environment such as limited access to education and healthcare [27, 28], as well as exposure to high levels of community violence [29]. It also has been suggested that adolescents facing stressors related to living in high poverty settings may experience feelings of hopelessness, helplessness, and social isolation [30]. Overall, all these risk factors may interact and compound each other and as a result, living in impoverished conditions has been linked to increased risks of internalising symptoms and mental health conditions in adolescents [7, 13, 17]. However, there is a need to better understand which of the risk factors described above may be the key drivers of such associations. This understanding is crucial to inform timely and targeted interventions that can effectively address the mental health challenges faced by adolescents living in high risk contexts in LMICs, and to inform the design of preventative intervention rather than interventions after the problems arise. Variable-selection approaches, based on current available literature, may be particularly suitable for such an undertaking, as they identify the most important predictors across a set of correlated variables, as in the current case.

Of note, while recent exposures (i.e., during adolescence) often show larger predictive effects, early childhood has been proposed to be a key developmental phase during which a child develops critical cognitive abilities, emotional regulation skills, and attachment through interactions with their parents or caregivers [31, 32]. Early childhood development has been shown to be affected by poverty and associated risk factors [31], which can have lasting negative effects throughout the life course [31, 33], including on mental health [34]. To design effective targeted interventions, it is thus of interest to investigate how predictors during early childhood influence the development of internalising symptoms in adolescence, and whether they have unique predictive effects above and beyond factors assessed later in life (in this case for adolescence).

The selection of early adolescent predictors was guided by existing literature on risk and protective factors for adolescent mental health in similar contexts. Variables such as house type (formal vs. informal housing), caregiver depression, caregiver alcohol use, exposure to violence, and food insecurity were chosen as they are well-established socioeconomic and environmental risk factors prevalent in high-risk, low-income setting. In addition, protective factors, including family support and friend/peer support were included to capture the influence of social relationships on mental health outcomes. Finally, self-esteem was included as an important psychological variable known to be a strong protective factor against the development of internalising symptoms in adolescents.

In summary, the current study aims to apply an elastic net variable-selection approach, in order to identify key early childhood and early adolescent predictors of internalising symptoms amongst 16 to 19 year-old adolescents in a high-risk LMIC context. It utilises data from a longitudinal study conducted in a South African informal settlement and includes data on early childhood predictors (antenatally to 18 month postpartum) and early adolescent predictors (13 to 14 years).

## Methods

### Setting

This longitudinal study took place in Khayelitsha, which is the fastest growing and largest township in the Western Cape, South Africa [35]. The estimated population of Khayelitsha is between 400 000 and 750 000, of which 99% are of Black African origin [36–38]. The township has the largest concentration of informal settlements in Cape Town and consists of a combination of formal (i.e., brick housing) and informal areas (i.e., shacks or temporary housing) [36, 37]. Half of Khayelitsha’s population falls within the poorest income quintile for Cape Town and it has an unemployment rate of between 38 and 74% [39, 40]. Khayelitsha also has the highest prevalence rate of HIV infections in the Western Cape [35], and the area is characterised by significantly high levels of crime and lack of consistent and effective service delivery and police services [41].

## Sample and procedures

Over a period of almost two decades, participants in this study were involved in a research project consisting of three phases. During Phase 1, conducted from 1999 to 2003, pregnant women in their third trimester were recruited to participate in a randomized controlled trial called “Thula Sana,” which aimed to improve maternal sensitivity and responsiveness [42]. During Phase 1, 449 pregnant women were enrolled into the study and assessed across five time-points from the antenatal period to 18 months post-partum. These five time-points were: antenatally (*n* = 449); 2 months follow-up (*n* = 395–89%); 6 months follow-up (*n* = 354–79%); 12 months follow-up (*n* = 346–77%); and 18 months follow-up (*n* = 342–76%). Mothers randomised to the intervention group received the Thula Sana intervention from the third trimester up until their infants were 6 months old. Mothers randomised to the control group received treatment as usual, which were government-delivered antenatal and maternity services. A significant beneficial effect on the mother-infant relationship was observed [42].

**Fig. 1:**
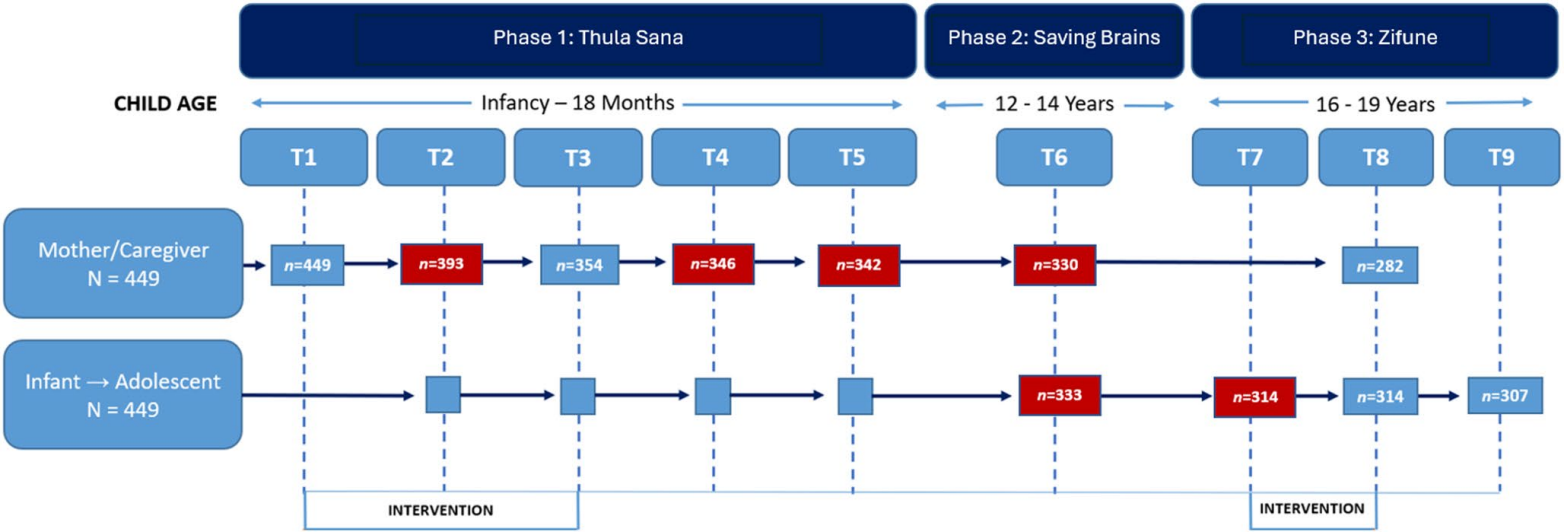
Longitudinal study time-points. Time-points included in this study are coloured in red. T1 (Antenatal); T2 (2 months follow-up); T3(6 months follow-up); T4(12 months follow-up); T5 (18 months follow-up); T6 (12–14 years follow-up); T7-T9 (16–19 years follow-up

Phase 2 of the study, conducted between 2012 and 2014, was part of the “Saving Brains” initiative. A total of 333 (74%) children, between the ages of 13–14 years, along with their primary caregiver (*n* = 330), were re-enrolled into the study to investigate the potential long-term beneficial effects of the Thula Sana intervention. Children were assessed on socio-emotional and cognitive functioning. No significant long-term impact on child development was observed. However, a positive effect on caregiver depression symptoms were observed [43]. Most recently, in 2018, during Phase 3 of the study, the children, now adolescents between the ages of 16 and 19 years, were re-enrolled and re-randomised into a new intervention trial called “Zifune”. Zifune aimed to evaluate the effect of a second-wave intervention to prevent violence amongst adolescents. During this phase of the study, adolescents were assessed during baseline (*n* = 314); post-intervention follow-up (*n* = 314); and 3-month follow-up (*n* = 307); and their primary caregivers (*n* = 282) at one time-point. Primary outcome data on internalising symptoms were collected during the Phase 3 baseline assessment. Exposure to the Thula Sana intervention (delivered during Phase 1) were controlled for. Figure 1provide an overview of the longitudinal study.

To assess early childhood predictors of internalising symptoms amongst adolescents, data collected during three-time points (2-month follow-up, 12-month follow-up, and 18-month follow-up) during Phase 1 of the study were used. To assess early adolescent predictors, data collected during Phase 2 of the study, from adolescents and their caregivers, were used.

Ethical approval granted across the three phases of the project are as following: Phase 1 (University of Reading [ref: 99/20] and the University of Cape Town [ref: 180/97]); Phase 2 (Health Research Ethics Committee at Stellenbosch University [S12/04/113]); and Phase 3 (Health Research Ethics Committee at Stellenbosch University [N17/10/094]).

## Measures

### Sociodemographic factors

The following sociodemographic factors were considered in the analysis (measured both, at 12 months of age and 13 years): child sex (sex assigned at birth), housing type (formal housing vs. informal housing); number of household members, caregiver employment status (any employment versus no employment), and household monthly income (only available for Phase 2 of the study).

## Early childhood factors

### Infant attachment style

The infants’ attachment style was assessed at 18 months using the Strange Situation procedure developed by Ainsworth and colleagues [44]. This standardised assessment procedure included having the infant filmed through a one-way mirror, in an unfamiliar playroom, over a period of 21 min. During this period the infant underwent standardised episodes of separation and reunion with the mother in the presence of a stranger. Video recordings were then independently rated by a blinded reviewer to assess infants as securely or insecurely attached [42]. To establish inter-rater reliability, a subset of the videotapes was scored by a second trained rater that was also blinded to treatment conditions to confirm inter-rater agreement (*k* = 0.96) [42].

## Maternal sensitivity

To assess the quality of mother-child interaction, two episodes of structured play situations were recorded at 12 months of child age. During each of these three-minute episodes, mothers were asked to play with their infant by using novel toys (a form board and stacking rings) [42]. A rater, blinded to the treatment group, then scored the behaviour of the mother from the videotapes [42]. To establish inter-rater reliability, a subset of the videotapes was scored by a trained rater that was also blinded to treatment conditions. An established measure to rate sensitivity on a five point scale was used [45, 46]. Sensitivity (comprising maternal expression of warmth, acceptance, and positive statements) was rated on a 5-point Likert-scale and summed across both tasks. Inter-rater reliability was established across 20 videotapes (*k* = 0.91).

## Maternal depression

Maternal depression was assessed at 2 months post-partum by a trained isiXhosa speaking researcher through using the major depression section of the clinical interview for Diagnostic and Statistical Manual of Mental Disorders, fourth edition (DSM-IV) [47]. Following standard translation principles, the interview was translated and then back translated to isiXhosa [48]. Recorded interviews and interviewer notes were then reviewed and assessed by the interviewer and a clinical psychologist, both blinded to treatment group [42]. A joint decision was then made regarding the presence of each of the relevant symptoms, which formed the basis for determining the presence or absence of a DSM-IV major depressive disorder [42, 47].

### Early adolescent factors

#### Food insecurity

Food insecurity was assessed through the 9-item Household Food Insecurity Access Scale (HFIAS) administered to adolescents [49]. The HFIAS uses a recall period of four weeks (30 days) to capture perceptions of food security, including anxiety about food supply, perceptions of the variety and quality of diet and insufficient food availability. The total score range for the HFIAS is 0–27, with higher scores indicating higher levels of food insecurity. The HFIAS has previously been used in studies assessing food insecurity in South Africa [50–52]. The HFIAS has been validated in a SSA country, showing good internal consistency (α = 0.90) [53].

### Family support

Adolescent perceived family support was assessed through the Multidimensional Scale of Perceived Social Support (MSPSS) scale [54]. The MSPSS is a 12-item scale and consists of three subscales assessing different sources of social support. The family support subscale consists of four items capturing perceived social support from family. The total score ranged from 4 to 12, with higher scores indicating higher levels of family support. The psychometric properties of the MSPSS (α = 0.86) have been demonstrated in a South African adolescent sample [55].

### Friend support

Friend support as perceived by the adolescent was assessed through the MSPSS [54]. The friend support subscale of the MSPSS consists of 4 items and follows the same procedure mentioned above (under ‘family support’).

### Self-esteem

Adolescents’ self-esteem was assessed through the 42-item Self-esteem Questionnaire (SEQ) [56]. Participants rated their agreement with items on six dimensions of self-esteem: peer relations, family, school, sport/athletics, body image, and global feelings of self-worth. The total score range is 42–168, with higher scores indicate higher levels of self-esteem. The SEQ has been previously validated in a South African adolescent population with adequate reliability for the majority of the subscales (α between 0.75 and 0.85 except for sport/athletics) [57].

### Exposure to violence

Violence exposure was rated by the adolescents through the Child Exposure to Community Violence (CECV) [58]. A shortened version (27-items) of the CECV adapted for the South African population was used, which captures the frequency of both direct and indirect exposure to domestic and neighbourhood violence common in this context [59]. The total score ranges from 0 to 52, with higher score indicating higher levels of violence exposure. The CECV shows good internal consistency (current sample α = 0.83) and is robustly associated with adolescent PTSS [60].

### Caregiver alcohol use

Caregiver alcohol use was assessed through questions asking the caregiver about the frequency of their alcohol consumption over the past month. Those caregivers that indicated that in the past month have consumed three or more drinks in a single day, were then administered the TWEAK. The TWEAK test, an acronym for Tolerance, Worried, Eye-opener, Amnesia, and Kut-down (Cut down), is a short 5-item screener for harmful alcohol use [61]. The psychometric properties of the TWEAK have not been assessed in a South African population. However, the TWEAK has been previously used to assessed alcohol consumption in South African studies [62, 63].

### Caregiver stress

The Parental Stress Index Short Form (PSI-SF) was used to assess caregiver stress. The PSI-SF comprises of three subscales capturing different parent-child problem areas: (1) parental distress; (2) parent-child dysfunctional interactions; (3) difficult child. Total scores range from 36 to 180, with lower scores indicating higher levels of caregiver stress. The PSI-SF has previously been found to have good internal consistency for the parental distress subscale (α = 0.75), the parent-child dysfunctional interactions subscale (α = 0.85), and the difficult child subscale (α = 0.82) [64].

### Caregiver depression symptoms

Caregiver depression symptoms were measured through the Patient Health Questionnaire (PHQ-9), which is a brief screening tool for depression [65]. Caregivers were asked to rate how often, over the past two weeks, they had been bothered by a range of symptoms of depression. Items are scored on a scale of 0–3 (0 = not at all; 1 = several days; 2 = more than half the days; 3 = nearly every day). The PHQ-9 has been extensively used and validated in several South African studies [66–68].

### Statistical analysis

All analyses were conducted in R version 4.3.0. In a first step, missing data patterns (for a summary, see supplementary file 1) resulting from participants dropping in and out of the study throughout their lifetime were addressed using the Multiple Imputation by Chained Equations (mice) algorithm, as implemented in the R package mice [69]. The algorithm iteratively imputes missing values by building predictive models for each incomplete variable using all other available variables in the dataset.

We then used the miselect package [70] to conduct an adaptive elastic net regularised regression to examine the effects of 18 early childhood and adolescence predictors on internalising symptoms at age 16 to 19 years. The elastic net is a regularisation technique that combines both the L1 (Lasso) and L2 (Ridge) penalties to perform variable selection amongst multiple correlated variables, with the aim of avoiding overfitting and controlling model complexity [71]. The adaptive elastic net approach extends the traditional elastic net by adapting the regularisation parameters for each predictor to achieve better prediction accuracy [72]. We used a gaussian link function and a stacked approach to combine estimates across the imputed datasets. For selecting tuning parameters, we performed a 5-fold cross-validation and selected the models with the lowest mean squared error (MSE). We also derived an approximate index of R-squared based on the MSE (R-squared = 1-(MSE/Var(Y)).

In order to account for the uncertainty associated with parameter estimation using the elastic net, we applied bootstrapping to the entire estimation procedure. We created 200 samples with replacement, and for each sample performed 20 multiple imputations. Stacked estimates for predictors, associated confidence intervals and the MSE/R-squared metric were pooled across bootstrapped datasets.

## Results

### Sociodemographic characteristics

The sociodemographic characteristics of the adolescent sample, collected during Phase 3 of the longitudinal study, are provided in Table 1. A total of 314 adolescent participants were included in the analysis. The mean age of adolescents was 17.20 years (SD = 0.64), with 53% being female. A total of 157 (50%) of adolescents had participated in the Thula Sana intervention as infants along with their mothers. A total of 25% of adolescents lived in informal housing. More than half of the population (59%) reported having repeated one or more school grades, and 10% reported leaving school prior to completion.

**Table 1:** Sociodemographic characteristics of adolescents

Sociodemographic characteristics	*N*	Percentage	Mean	SD
Adolescent age (years)	314		17.2	0.6
RCT (Thula Sana intervention)	157	50%		
Adolescent sex				
Male	148	47%		
Female	166	53%		
House type				
Formal housing	236	75%		
Informal housing	78	25%		
Living circumstances				
Running water at home	258	82%		
Flush toilet in home/on premises	227	72%		
Electricity at home	306	97%		
Biological mother is primary caregiver	264	84%		
Household member count			4.9	1.9
Household food insecurity^A^			7.2	5.7
Household monthly income^B^				
R0 - R499	11	4%		
R500 - R1000	20	6%		
R1001 - R2000	49	16%		
R2001 - R5000	115	37%		
R5001 - R8000	42	13%		
> R8000	41	13%		
* Data not available*	36	11%		
Education				
Currently enrolled	262	83.5%		
Correct grade for age	128	49.0%		
Finished school	20	6.5%		
Left school	32	10.0%		
Repeated grade	186	59.0%		

^A^ Score range from 0–27; Higher scores indicate higher levels of food insecurity; ^B^ Data not available for full sample

#### Early childhood and early adolescent predictors

Table 2 provides an overview of early childhood and early adolescent scores for each risk factor prior to multiple imputation. It highlights relatively high rates of poverty, with 79% of children living in informal housing at 2 months postpartum and 44% in adolescence, with high rates of maternal unemployment and 93% of household having a monthly income of ≤ 8000 ZAR at child aged 13 years. Table 2 shows the mean internalising symptoms score for the adolescent sample during Phase 3, which was 18.22 (SD = 8.75; range: 0–62). See Supplementary file 2 for the Person correlation coefficients between predictors at early childhood (EC) and early adolescence (EA).

**Table 2:** Predictors and outcomes

	Range	*N*	Percentage	Mean	SD
Early childhood predictors					
House type^A^					
Formal housing		59	21.0%		
Informal housing		227	79.0%		
Household member count ^A^				4.7	1.8
Caregiver employment^A^					
Employed/Self-employed		57	20.0%		
Unemployed		229	80.0%		
Infant attachment^C^					
Secure attached		167	66.0%		
Insecure attached		85	34.0%		
Maternal sensitivity^B^	2–10			5.6	1.8
Maternal depression^A^					
Depressed		71	25.0%		
Not depressed		217	75.0%		
Early adolescent predictors^D^					
House type					
Formal housing		162	56.0%		
Informal housing		127	44.0%		
Household member count				3.7	1.9
Household monthly income		288			
R0 – R499		4	1%		
R500 – R1000		36	13%		
R1001 - R2000		72	25%		
R2001 – R5000		120	41%		
R5001 – R8000		37	13%		
> R8000		19	7%		
Household food insecurity	0–27			9.91	5.9
Family support	4–12			11.6	0.9
Friend support	4–12			8.9	2.4
Self-esteem	42–168			131.7	11.8
Exposure to violence	0–52			6.9	5.4
Caregiver alcohol use	0–10			1.1	2.2
Caregiver stress	36–180			123.4	19.9
Outcomes^E^					
Internalising symptoms Total Score	0–62			18.32	8.75

^A^ Assessed at 2-month follow-up (Phase 1); ^B^ Assessed at 12-month follow-up (Phase 1); ^**C**^ Assessed at 18-month follow-up; ^**D**^ Assessed during Phase 2; ^E^ Assessed during Phase 3;

### Elastic net results

**Fig. 2:**
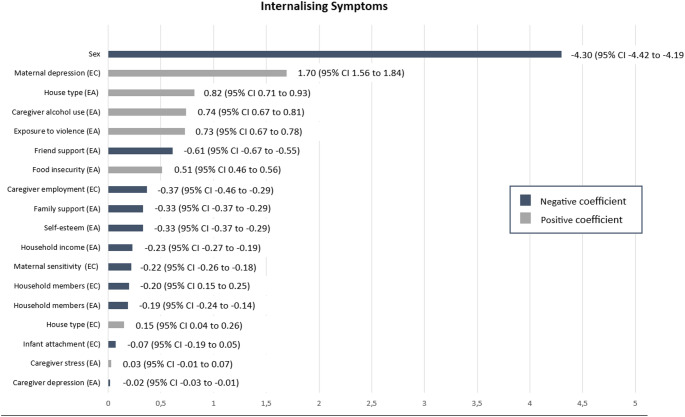
Elastic net results for internalising symptoms

Figure 2 reports on the elastic net results for internalising symptoms, ranked by their coefficient size. The approximation of the R2 value that we calculated based on the MSE indicated that 22% of the variance was explained on average across the boostrapped datasets (hyperparameters: α = 0.85, λ = 0.03, RMSE = 59.53).

The top 10 factors associated with higher levels of internalising symptoms were female sex (*β* = −4.30; 95% CI [−4.42; −4.19]), higher levels of maternal depression during early childhood (*β* = 1.70; 95% CI [1.56; 1.84]), living in a formal house during early adolescence (*β* = 0.82; 95% CI [0.71; 0.93]), higher levels of caregiver alcohol use during early adolescence (*β* = 0.74; 95% CI [0.67; 0.81]), exposure to higher levels of family and community violence during early adolescence (*β* = 0.73; 95% CI [0.67; 0.78]), lower levels of friend support during early adolescence (*β* = −0.61; 95% CI [−0.67; −0.55]), higher levels of food insecurity during early adolescence (*β* = 0.51; 95% CI [0.46; 0.56]), lack of caregiver employment during early childhood (*β* = −0.37; 95% CI [−0.46; −0.29]), lower levels of family support during early adolescence (*β* = −0.33; 95% CI [−0.37; −0.29]), and lower levels of self-esteem during early adolescence (*β* = −0.33; 95% CI [−0.37; −0.29]).

## Discussion

This study aimed to identify the early childhood and early adolescent predictors of internalising symptoms amongst older adolescents (16 to 19 years old) living in a high-risk LMIC context characterised by poverty and associated secondary risks. Utilising data collected as part of a longitudinal study, with 18 years of follow up, we found that two variables from early childhood (maternal depression and, caregiver employment) and seven during early adolescent (house type, caregiver alcohol use, exposure to violence, friend support, food insecurity, family support, and self-esteem) were amongst the strongest predictors of internalising symptoms.

Amongst all predictors that were assessed, female sex emerged as a key predictor of internalising symptoms. This finding was not surprising, as multiple previous studies have found that internalising symptoms are more common amongst females than males during adolescence [73–76]. While biological factors, such as hormonal changes during puberty, play a role, their effect is often compounded by gendered socialisation and environmental factors that often disproportionately affects girls in high-risk settings like those in South Africa [76–78]. Adolescent girls in South Africa often face a complex network of intersecting vulnerabilities, including a higher burden of unpaid domestic work and caregiving responsibilities within the household [79]. These gender roles often limit their access to educational opportunities and inflate their sense of responsibility, leading to increased stress and psychosocial strain. Furthermore, gender inequality in South Africa contributes to alarming rates of gender-based violence (GBV), with the country having some of the highest rates of intimate partner violence and femicide in the world [80].

Beyond this, three types of risk variables emerged among the largest predictors: Firstly, parental mental health and alcohol use. During early childhood, maternal depression emerged as an important predictor of higher levels of internalising symptoms during adolescence. Studies have identified that exposure to a depressed caregiver can have significant and lasting effects on child and adolescent mental health [81, 82]. A depressed caregiver may often display less maternal responsiveness or sensitivity, and more intrusive behaviour towards their child [82], which may be particularly detrimental for early childhood development [83]. While there is some evidence that such associations are found in LMICs [82], findings from this study contributes to the understanding that exposure to a depressed caregiver can have lasting effects on adolescent mental health in an environment characterised by compounded risks associated with poverty.

Furthermore, current caregiver alcohol use during early adolescence predictor associated with increased internalising symptoms. Previous studies have suggested that caregiver alcohol use can result in emotional neglect, inconsistent parenting, and family dysfunction [84–86]. These factors can create a stressful and unstable home environment, which is known to be detrimental to adolescent mental health [86]. Therefore, alcohol interventions and management must be a priority and follow a multi-faceted approach that addresses both the individual challenges faced by caregivers and the broader social and economic factors that contribute to increased substance use.

A few predictors related to poverty, including house type, and other secondary risks, were associated with internalising symptoms. Living in an informal house serves as an indicator of poverty, and it can also act as a proxy for other compounding risk factors. symptoms. Adolescents in such environments are exposed to environmental instability (e.g., inconsistent access to electricity, sanitation, and clean water) and social stressors (e.g., overcrowding in homes and lack of privacy).

Also, caregiver employment during early childhood emerged as a predictor, suggesting the potential protective role of a stable environment within the family. Stable employment may contribute to a nurturing, supportive, and stable environment for a child [87, 88]. The importance of employment should be considered not only for its economic benefits but also for its potential to contribute to a sense of accomplishment, which can positively impact household functioning [89]. These findings highlight the significance of socio-economic factors in affecting internalising symptoms and emphasises the need for policies and interventions that supports families’ financial stability as research has shown that financial stability can be associated with fewer mental health problems during adolescence [90–92].

Food insecurity during early adolescence was found to be associated with higher levels of internalising symptoms. Insufficient access to adequate and nutritious food is often a secondary consequence of living in poverty and parental unemployment and can have a profound impact on the development and mental health of a child and adolescent [93–96]. Most studies assessing the effect of food insecurity on adolescent mental health are limited to cross-sectional analysis [95, 97]. The current finding contributes to our understanding of how food insecurity can affect adolescent mental health over time, which is especially relevant in the context of South Africa, where almost 20% of households have inadequate or severely inadequate access to food [98]. Implementing comprehensive strategies such as increasing access to adequate food, improving economic conditions, enhancing employment, pay standards and cash grants, and promoting community-based support services can mitigate against the negative effects of food insecurity on adolescent mental health [99, 100].

Exposure to violence in the community and home also emerged as a predictor of high levels of internalising symptoms. This is in accordance with previous research indicating that violence exposure predicts higher levels of mental health problems, including internalising symptoms [96–103]. Nearly 70% of adolescents in South Africa report having witnessed or been victims of violence [101], with many experiencing multiple forms simultaneously [102]. Improving violence prevention and providing appropriate support to individuals affected by violence are critical steps towards promoting mental well-being in adolescents. As such, school-based interventions, community programmes, and mental health services can play a crucial role in creating safe environments and helping adolescents cope with exposure to violence [103, 104].

Family and friend/peer support both emerged as predictors of lower levels of internalising symptoms, indicating their potential protective role against such symptoms. While these factors are universally recognised as important [105–108], their influence is particularly critical in contexts marked by high rates of poverty, unemployment and violence. In environments with limited social safety nets, family and peer support can serve as a budder against stress and trauma [109]. Family support which emerged as a significant predictor, can provide a sense of security and belonging that can help adolescents cope with daily challenges [110]. Similarly, strong peer support fosters resilience by creating a safe space where adolescents can share experiences, feel a sense of belonging, and receive mutual encouragement [111].

A healthy self-esteem also emerged as a protective factor against internalising symptoms. Previous studies have also reported on the significance of self-esteem in relation to internalising symptoms [112] suggesting it acts as a buffer against the negative or adverse effects on mental health [113, 114]. However, it is of interest to note that in this context characterised by high deprivation, such commonly variables commonly found relevant in HIC contexts play a comparatively smaller role than poverty and other psychosocial risks.

Overall, the current findings highlight the importance of considering the cumulative impact of multiple risk factors, over time, in understanding and addressing internalising symptoms. This study provides insight into how different individual, social, and environmental factors affect internalising symptoms across different life stages, in a high-risk population. Interventions that target specific risk factors in isolation may not be as effective as those that adopt a holistic approach, considering the interrelationship among these factors. A recent South African study provided evidence that addressing multiple factors simultaneously (i.e., safe communities, food security, and government cash transfers) can lead to better mental health outcomes in adolescents [115]. However, outcome studies evaluating the effectiveness of interventions targeting multiple factors simultaneously, instead of single factors, remains limited [116].

### Limitations

First, this study utilised a small sample size, that is on the lower end of what is recommended for the usage of selection approaches such as the elastic net. It also meant that we were unable to perform a test-training split to validate the predictiveness of our model in a separate sample. As a result, we focused predominantly on inference and not predictions but would recommend future studies to replicate the current findings with larger samples. Second, we were somewhat limited by currently available packages to perform both multiple imputation and variable selection, and were as such not able to set up nested k-fold cross-validation, which is more suitable for small samples [117]. Future studies should aim to address this by expanding current methods or adopting a full Bayesian approach. Third, we only looked at internalising problems in a broad manner. Future studies may explore pathways towards specific disorders (e.g., depression, anxiety, post-traumatic stress disorder). Fourth, ideally, we would have wanted more intermediate measures between early childhood and early adolescent outcomes in order to investigate potential pathways through which the selected predictors can potentially affect internalising symptoms. It is also important to note that the group were exposed to two interventions over time – the primary reason for the study initially. Although exposure to such interventions were controlled for, the interventions themselves may have impacted on the findings and the functioning of at least some of the participants. This would suggest that when generalising to the broader population who have not been exposed to trial interventions, the situation may be different – perhaps worse.

## Supplementary Information

Below is the link to the electronic supplementary material.

Supplementary Material 1

Supplementary Material 2

## Data Availability

No datasets were generated or analysed during the current study.

## References

[1] World Health Organization (2017) *Global Accelerated Action for the Health of Adolescents (AA-HA!): guidance to support country implementation.*

[2] World Health Organization. Mental disorders (2022) 8 June 2022 24 March 2023]; Available from: https://www.who.int/news-room/fact-sheets/detail/mental-disorders

[3] Najman JM et al (2010) Family poverty over the early life course and recurrent adolescent and young adult anxiety and depression: a longitudinal study. Am J Public Health 100(9):1719–1723 20634459 10.2105/AJPH.2009.180943 PMC2920957

[4] Dashiff C et al (2009) Poverty and adolescent mental health. J Child Adolesc Psychiatric Nurs 22(1):23–32 10.1111/j.1744-6171.2008.00166.x 19200289

[5] Iemmi V et al (2016) Suicide and poverty in low-income and middle-income countries: a systematic review. Lancet Psychiatry 3(8):774–783 27475770 10.1016/S2215-0366(16)30066-9

[6] Dupéré V, Leventhal T, Lacourse E (2009) Neighborhood poverty and suicidal thoughts and attempts in late adolescence. Psychol Med 39(8):1295–1306 18845013 10.1017/S003329170800456X

[7] Fang M (2018) School poverty and the risk of attempted suicide among adolescents. Soc Psychiatry Psychiatr Epidemiol 53:955–967 29947861 10.1007/s00127-018-1544-8

[8] Eamon MK (2002) Influences and mediators of the effect of poverty on young adolescent depressive symptoms. J Youth Adolesc 31:231–242

[9] McBride Murry V et al (2011) Neighborhood poverty and adolescent development. J Res Adolescence 21(1):114–128

[10] Compas BE (2004) *Processes of risk and resilience during adolescence: Linking contexts and individuals.* Handbook of adolescent psychology, : pp. 263–296

[11] Hodgkinson S et al (2017) Improving mental health access for low-income children and families in the primary care setting. Pediatrics. 10.1542/peds.2015-1175 27965378 10.1542/peds.2015-1175 PMC5192088

[12] Evans GW, Kim P (2013) Childhood poverty, chronic stress, self-regulation, and coping. Child Dev Perspect 7(1):43–48

[13] Erskine H et al (2017) The global coverage of prevalence data for mental disorders in children and adolescents. Epidemiol Psychiatric Sci 26(4):395–402 10.1017/S2045796015001158 PMC6998634 26786507

[14] Das-Munshi J et al (2016) Mental health inequalities in adolescents growing up in post-apartheid South africa: Cross-sectional survey, SHaW study. PLoS ONE 11(5):e0154478 27139456 10.1371/journal.pone.0154478 PMC4854374

[15] Patel V et al (2007) Mental health of young people: a global public-health challenge. Lancet 369(9569):1302–1313 17434406 10.1016/S0140-6736(07)60368-7

[16] North A et al (2020) Pathways from witnessing community violence to mental health problems among South African adolescents. South Afr Med J 110(2):145–153 10.7196/SAMJ.2020.v110i2.13929 PMC9327528 32657687

[17] Tien J, Lewis GD, Liu J (2020) Prenatal risk factors for internalizing and externalizing problems in childhood. World J Pediatr 16:341–355 31617077 10.1007/s12519-019-00319-2 PMC7923386

[18] Cabral MD, Patel DR (2020) *Risk factors and prevention strategies for anxiety disorders in childhood and adolescence.* Anxiety Disorders: Rethinking and understanding recent discoveries, : pp. 543–559 10.1007/978-981-32-9705-0_27 32002945

[19] Narmandakh A et al (2021) Psychosocial and biological risk factors of anxiety disorders in adolescents: a TRAILS report. Eur Child Adolesc Psychiatry 30:1969–1982 33113027 10.1007/s00787-020-01669-3 PMC8563629

[20] Dumont IP, Olson AL (2012) Primary care, depression, and anxiety: exploring somatic and emotional predictors of mental health status in adolescents. J Am Board Fam Med 25(3):291–299 22570392 10.3122/jabfm.2012.03.110056

[21] Pelkonen M, Marttunen M (2003) Child and adolescent suicide: epidemiology, risk factors, and approaches to prevention. Pediatr Drugs 5:243–265 10.2165/00128072-200305040-00004 12662120

[22] Kandel DB, Raveis VH, Davies M (1991) Suicidal ideation in adolescence: depression, substance use, and other risk factors. J Youth Adolesc 20(2):289–309 24265011 10.1007/BF01537613

[23] Wight V et al (2014) *Understanding the Link between Poverty and Food Insecurity among Children: Does the Definition of Poverty Matter?* J Child Poverty 20(1):1–20 25045244 10.1080/10796126.2014.891973 PMC4096937

[24] Hatem C et al (2020) Food insecurity and housing instability during early childhood as predictors of adolescent mental health. J Fam Psychol 34(6):721–730 32191051 10.1037/fam0000651 PMC7483158

[25] Hall BJ et al (2019) Perspectives of adolescent and young adults on poverty-related stressors: a qualitative study in Ghana, Malawi and Tanzania. BMJ Open 9(10):e027047 31615792 10.1136/bmjopen-2018-027047 PMC6797331

[26] Manhica H et al (2021) Association between poverty exposure during childhood and adolescence, and drug use disorders and drug-related crimes later in life. Addiction 116(7):1747–1756 33197093 10.1111/add.15336 PMC8247994

[27] Tessema ZT et al (2022) Determinants of accessing healthcare in Sub-Saharan africa: a mixed-effect analysis of recent demographic and health surveys from 36 countries. BMJ Open 12(1):e054397 35105635 10.1136/bmjopen-2021-054397 PMC8804632

[28] Nortje MJ (2017) The effect of poverty on education in South Africa. Educor Multidiscip J 1(1):47–62

[29] Shields N, Nadasen K, Pierce L (2008) The effects of community violence on children in Cape Town, South Africa. Child Abuse Negl 32(5):589–601 18511114 10.1016/j.chiabu.2007.07.010

[30] Landis D et al (2007) Urban adolescent stress and hopelessness. J Adolesc 30(6):1051–1070 17467052 10.1016/j.adolescence.2007.02.001

[31] Black MM et al (2017) Early childhood development coming of age: science through the life course. Lancet 389(10064):77–90 27717614 10.1016/S0140-6736(16)31389-7 PMC5884058

[32] Maggi S et al (2010) The social determinants of early child development: an overview. J Paediatr Child Health 46(11):627–635 20796183 10.1111/j.1440-1754.2010.01817.x

[33] Treanor M (2012) Impacts of poverty on children and young people. Scottish Childcare and Protection Network Research Briefing, Edinburgh

[34] Yoshikawa H, Aber JL, Beardslee WR (2012) The effects of poverty on the mental, emotional, and behavioral health of children and youth: implications for prevention. Am Psychol 67(4):272 22583341 10.1037/a0028015

[35] Stinson KG, Coetzee E, Van Cutsem D (2017) Gilles. Hilderbrand, Katherine. Osler, Meg. Hennessey, Claudine. Wilkinson, Lynne. Patten, Gabriela. Cragg, Carol. Mathee, Shaheed, Cox, Vivian. Boulle, Andrew, *Cohort profile: the Khayelitsha antiretroviral programme, cape Town, South Africa*. Int J Epidemiol 46(2):e21 27208042 10.1093/ije/dyw057

[36] *South African Census 2011: Khayelitsha*. 2011 [cited 2023 20 January]; Available from: http://census2011.adrianfrith.com/place/199038

[37] *Khayelitsha*. (2022) [cited 2023; Available from: https://en.wikipedia.org/wiki/Khayelitsha

[38] Super G (2015) *Violence and democracy in Khayelitsha, governing crime through the ‘community’.* Stability: International Journal of Security and Development, 4(1)

[39] City of Cape Town (2013) *2011 Census – Khayelitsha Health District*

[40] Seekings J (2013) *Economy, society and municipal services in Khayelitsha.* Report for the commission of inquiry into allegations of Police inefficiency in Khayelitsha and a breakdown in relations between the community and the Police in Khayelitsha. Centre for Social Science Research, University of Cape Town

[41] Freeman L, McDonald C (2015) Mapping Khayelitsha-the complexities of everyday policing in a high crime area. South Afr Crime Q 53:27–37

[42] Cooper PJ et al (2009) Improving quality of mother-infant relationship and infant attachment in socioeconomically deprived community in South Africa: randomised controlled trial. BMJ. 10.1136/bmj.b974 19366752 10.1136/bmj.b974 PMC2669116

[43] Tomlinson M et al (2022) First 1,000 days: enough for mothers but not for children? Long-term outcomes of an early intervention on maternal depressed mood and child cognitive development: follow‐up of a randomised controlled trial. J Child Psychol Psychiatry 63(3):261–272 34227113 10.1111/jcpp.13482

[44] Ainsworth M et al (1978) Patterns of attachment: a psychological study of the strange situation. Lawrence Erlbaum, New Jersey

[45] Wolke D, Skuse D, Mathisen B (1990) Behavioral style in failure-to-thrive infants: a preliminary communication. J Pediatr Psychol 15(2):237–254 2374078 10.1093/jpepsy/15.2.237

[46] Stein A et al (1994) An observational study of mothers with eating disorders and their infants. J Child Psychol Psychiatry 35(4):733–748 8040225 10.1111/j.1469-7610.1994.tb01218.x

[47] First M et al (1996) *User’s Guide for the SCID-IV. Structured clinical interview for DSM-IV axis 1 disorders.*

[48] Brislin RW (1986) *The wording and translation of research instruments.*

[49] Coates J, Swindale A, Bilinsky P (2007) *Household Food Insecurity Access Scale (HFIAS) for measurement of food access: indicator guide: version 3.*

[50] Musemwa L et al (2015) Household food insecurity in the poorest Province of South africa: level, causes and coping strategies. Food Secur 7(3):647–655

[51] Ndhleve S et al (2021) Household food insecurity status and determinants: the case of Botswana and South Africa. AGRARIS: J Agribusiness Rural Dev Res 7(2):207–224

[52] Shisanya S, Mafongoya P (2016) Adaptation to climate change and the impacts on household food security among rural farmers in uMzinyathi district of Kwazulu-Natal, South Africa. Food Secur 8(3):597–608

[53] Knueppel D, Demment M, Kaiser L (2010) Validation of the household food insecurity access scale in rural Tanzania. Public Health Nutr 13(3):360–367 19706211 10.1017/S1368980009991121

[54] Zimet G (2016) *Multidimensional Scale of Perceived Social Support (MSPSS) - Scale Items and Scoring Information*

[55] Bruwer B et al (2008) Psychometric properties of the multidimensional scale of perceived social support in youth. Compr Psychiatry 49(2):195–201 18243894 10.1016/j.comppsych.2007.09.002

[56] Dubois D et al (1996) Early adolescent self-esteem: A developmental-ecological framework and assessment strategy. J Res Adolescence 6:543–579

[57] Wild LG et al (2005) Psychometric properties of the self-esteem questionnaire for South African adolescents. South Afr J Psychol 35(2):195–208

[58] Amaya-Jackson L (1998) *Child’s exposure to violence checklist.* Adapted from Richter’s Things I’ve seen and heard

[59] Martin L, Revington N, Seedat S (2013) The 39-item child exposure to community violence (CECV) scale: exploratory factor analysis and relationship to PTSD symptomatology in trauma-exposed children and adolescents. Int J Behav Med 20(4):599–608 23055027 10.1007/s12529-012-9269-7

[60] Boyes ME, Cluver LD, Gardner F (2012) Psychometric properties of the child PTSD checklist in a community sample of South African children and adolescents. PLoS One. 10.1371/journal.pone.0046905 23056523 10.1371/journal.pone.0046905 PMC3463511

[61] Russell M (1994) New assessment tools for risk drinking during pregnancy: T-ACE, TWEAK, and others. Alcohol Health Res World 18(1):55 31798157 PMC6876474

[62] Dewing S et al (2013) Food insecurity and its association with co-occurring postnatal depression, hazardous drinking, and suicidality among women in peri-urban South Africa. J Affect Disord 150(2):460–465 23707034 10.1016/j.jad.2013.04.040 PMC3762324

[63] Rotheram-Borus MJ et al (2023) Maternal depression, alcohol use, and transient effects of perinatal paraprofessional home visiting in South africa: Eight-year follow-up of a cluster randomized controlled trial. Soc Sci Med 324:115853 37001280 10.1016/j.socscimed.2023.115853 PMC10121853

[64] Barroso NE et al (2016) Psychometric properties of the parenting stress Index-Short form (PSI-SF) in a high-risk sample of mothers and their infants. Psychol Assess 28(10):1331–1335 26595220 10.1037/pas0000257 PMC4877285

[65] Kroenke K, Spitzer RL (2002) The PHQ-9: a new depression diagnostic and severity measure. SLACK Incorporated Thorofare, NJ, pp 509–515

[66] Petersen I et al (2014) A group-based counselling intervention for depression comorbid with HIV/AIDS using a task shifting approach in South africa: a randomized controlled pilot study. J Affect Disord 158:78–84 24655769 10.1016/j.jad.2014.02.013

[67] Bhana A et al (2015) The validity of the patient health questionnaire for screening depression in chronic care patients in primary health care in South Africa. BMC Psychiatry 15(1):1–9 26001915 10.1186/s12888-015-0503-0 PMC4446842

[68] Cholera R et al (2014) Validity of the patient health questionnaire-9 to screen for depression in a high-HIV burden primary healthcare clinic in Johannesburg, South Africa. J Affect Disord 167:160–166 24972364 10.1016/j.jad.2014.06.003 PMC4264106

[69] Van Buuren S, Groothuis-Oudshoorn K, Robitzsch A (2019) Package ‘mice’: multivariate imputation by chained equations. CRAN Repos

[70] Du J et al (2022) Variable selection with multiply-imputed datasets: choosing between stacked and grouped methods. J Comput Graphical Stat 31(4):1063–1075 10.1080/10618600.2022.2035739 PMC9838615 36644406

[71] Zou H, Hastie T (2005) Regularization and variable selection via the elastic net. J R Stat Soc Ser B Stat Methodol 67(2):301–320

[72] Zou H, Zhang HH (2009) On the adaptive elastic-net with a diverging number of parameters. Ann Stat 37(4):1733 20445770 10.1214/08-AOS625 PMC2864037

[73] Durbeej N et al (2019) Trends in childhood and adolescent internalizing symptoms: results from Swedish population based twin cohorts. BMC Psychol 7(1):50 31375136 10.1186/s40359-019-0326-8 PMC6679471

[74] Angold A, Costello EJ, Worthman CM (1998) Puberty and depression: the roles of age, pubertal status and pubertal timing. Psychol Med 28(1):51–61 9483683 10.1017/s003329179700593x

[75] Thompson SM (2017) The role of puberty in the development of depressive symptoms into young adulthood. UCLA

[76] Van Droogenbroeck F, Spruyt B, Keppens G (2018) Gender differences in mental health problems among adolescents and the role of social support: results from the Belgian health interview surveys 2008 and 2013. BMC Psychiatry 18(1):6 29320999 10.1186/s12888-018-1591-4 PMC5763832

[77] McGuire TC et al (2019) Pubertal maturation and trajectories of depression during early adolescence. Front Psychol 10:1362 31244742 10.3389/fpsyg.2019.01362 PMC6582206

[78] Albert PR (2015) Why is depression more prevalent in women? J Psychiatry Neurosci 40(4):219–221 26107348 10.1503/jpn.150205 PMC4478054

[79] Inclusive Society Institute (2023) Understanding youth inequality. Cape Town,

[80] Mkwananzi S, Nathane-Taulela M (2024) Gender-based violence and femicide interventions-perspectives from community members and activists in South Africa. Front Glob Womens Health 5:1199743 39113900 10.3389/fgwh.2024.1199743 PMC11303172

[81] Campbell SB et al (2004) The course of maternal depressive symptoms and maternal sensitivity as predictors of attachment security at 36 months. Dev Psychopathol 16(2):231–252 15487594 10.1017/s0954579404044499

[82] Sevenoaks T et al (2022) A longitudinal and qualitative analysis of caregiver depression and quality of life in the cape town adolescent antiretroviral cohort. J Affect Disorders Rep 10:100396

[83] Bernard-Bonnin A-C et al (2004) Maternal depression and child development. Paediatr Child Health 9(8):575–583 19680490 10.1093/pch/9.8.575 PMC2724169

[84] Hughes K et al (2017) The effect of multiple adverse childhood experiences on health: a systematic review and meta-analysis. Lancet Public Health 2(8):e356–e366 29253477 10.1016/S2468-2667(17)30118-4

[85] Jacques DT et al (2020) Maternal alcohol dependence and harsh caregiving across parenting contexts: the moderating role of child negative emotionality. Dev Psychopathol 32(4):1509–1523 31735197 10.1017/S0954579419001445 PMC7231671

[86] Lander L, Howsare J, Byrne M (2013) The impact of substance use disorders on families and children: from theory to practice. Soc Work Public Health 28(3–4):194–205 23731414 10.1080/19371918.2013.759005 PMC3725219

[87] Association AP (2018) Children, youth, families and socioeconomic status. APA Fact Sheet

[88] Cooper K, Stewart K (2021) Does household income affect children’s outcomes? A systematic review of the evidence. Child Indic Res 14(3):981–1005

[89] Heinrich CJ (2014) Parents’ employment and children’s wellbeing. Future Child. 10.1353/foc.2014.0000 25518706 10.1353/foc.2014.0000

[90] Ridley M et al (2020) Poverty, depression, and anxiety: causal evidence and mechanisms. Science 370(6522):eaay0214 33303583 10.1126/science.aay0214

[91] Cluver L et al (2013) Child-focused state cash transfers and adolescent risk of HIV infection in South africa: a propensity-score-matched case-control study. Lancet Global Health 1(6):e362–e370 25104601 10.1016/S2214-109X(13)70115-3

[92] Sherr L et al (2017) Can cash break the cycle of educational risks for young children in high HIV–affected communities? A cross–sectional study in South Africa and Malawi. J Glob Health. 10.7189/jogh.07.020409 29302316 10.7189/jogh.07.020409 PMC5735773

[93] Slopen N et al (2010) Poverty, food insecurity, and the behavior for childhood internalizing and externalizing disorders. J Am Acad Child Adolesc Psychiatry 49(5):444–452 20431464 10.1097/00004583-201005000-00005

[94] Jyoti DF, Frongillo EA, Jones SJ (2005) Food insecurity affects school children’s academic performance, weight gain, and social skills. J Nutr 135(12):2831–2839 16317128 10.1093/jn/135.12.2831

[95] Poole-Di Salvo E, Silver EJ, Stein RE (2016) Household food insecurity and mental health problems among adolescents: what do parents report? Acad Pediatr 16(1):90–96 26530851 10.1016/j.acap.2015.08.005

[96] Alaimo K, Olson CM, Frongillo EA (2002) Family food insufficiency, but not low family income, is positively associated with dysthymia and suicide symptoms in adolescents. J Nutr 132(4):719–725 11925467 10.1093/jn/132.4.719

[97] Elgar FJ et al (2021) Food insecurity, state fragility and youth mental health: A global perspective. SSM Popul Health 14:100764 33732866 10.1016/j.ssmph.2021.100764 PMC7944102

[98] STATS SA (2019) *Towards Measuring the Extent of Food Security in South Africa: An Examination of Hunger and Food inadequacy*

[99] Durao S et al (2020) Community-level interventions for improving access to food in low- and middle-income countries. Cochrane Database Syst Rev, 7(7): p. Cd011504. 10.1002/14651858.CD011504.pub2 PMC7390433 32722849

[100] Mbajiorgu DG, Odeku KO (2022) Fighting food insecurity, hunger, and poverty: the content and context of the socio-economic right of access to sufficient food in South Africa. Obiter 43(3):467–488

[101] Distiller G et al (2007) Factors affecting resilience in children exposed to violence. South Afr J Psychol 37(1):165–187

[102] Kaminer D et al (2013) Exposure to violence across multiple sites among young South African adolescents. Peace Conflict J Peace Psychol 19(2):112

[103] Tomlinson M, Kleintjes S, Lake L (2022) South African Child Gauge 2021/2022. Children’s Institute, University of Cape Town

[104] Unicef (2022) *Promoting and protecting mental health in schools and learning environments.*

[105] Carbonell DM et al (2002) Adolescent protective factors promoting resilience in young adults at risk for depression. Child Adolesc Soc Work J 19:393–412

[106] Liu Y (2006) Paternal/maternal attachment, peer support, social expectations of peer interaction, and depressive symptoms. Adolescence, 41(164) 17240776

[107] La Greca AM, Harrison HM (2005) *Adolescent peer relations, friendships, and romantic relationships: do they predict social anxiety and depression?* J Clin Child Adolesc Psychol 34(1):49–61 15677280 10.1207/s15374424jccp3401_5

[108] Liem JH, Lustig K, Dillon C (2010) Depressive symptoms and life satisfaction among emerging adults: a comparison of high school dropouts and graduates. J Adult Dev 17:33–43

[109] Nyathi L et al (2024) Social issues affecting social cohesion in Low-resource communities in South Africa. Afr J Gov Dev 13(2):135–162

[110] Roman NV et al (2025) Strengthening family bonds: A systematic review of factors and interventions that enhance family cohesion. Social Sci 14(6):371

[111] Butler N et al (2022) The contributing role of Family, School, and peer supportive relationships in protecting the mental wellbeing of children and adolescents. School Ment Health 14(3):776–788 35154501 10.1007/s12310-022-09502-9 PMC8818094

[112] Ngo H, VanderLaan DP, Aitken M (2020) Self-esteem, symptom severity, and treatment response in adolescents with internalizing problems. J Affect Disord 273:183–191 32421601 10.1016/j.jad.2020.04.045

[113] Auttama N et al (2021) Factors associated with Self-Esteem, Resilience, mental Health, and psychological Self-Care among university students in Northern Thailand. J Multidiscip Healthc 14:1213–1221 34079280 10.2147/JMDH.S308076 PMC8166326

[114] Henriksen IO et al (2017) The role of self-esteem in the development of psychiatric problems: a three-year prospective study in a clinical sample of adolescents. Child Adolesc Psychiatry Ment Health 11:68 29299058 10.1186/s13034-017-0207-y PMC5747942

[115] Mebrahtu H et al (2022) Can a combination of interventions accelerate outcomes to deliver on the sustainable development goals for young children? Evidence from a longitudinal study in South Africa and Malawi, vol 48. Care, Health and Development, Child, pp 474–485. 3 10.1111/cch.12948 34907593

[116] Knight A et al (2017) The quality and effectiveness of interventions that target multiple risk factors among young people: a systematic review. Aust N Z J Public Health 41(1):54–60 27624886 10.1111/1753-6405.12573 PMC5298033

[117] Vabalas A et al (2019) Machine learning algorithm validation with a limited sample size. PLoS ONE 14(11):e0224365 31697686 10.1371/journal.pone.0224365 PMC6837442

