# Supplementary material for: Early childhood and early adolescent predictors of internalising symptoms in adolescents: findings from a longitudinal study in a high-risk South African environment
Source: Soc Psychiatry Psychiatr Epidemiol. 2026 Feb 11;61(7):1293–304. doi: 10.1007/s00127-026-03048-w (PMC13424707; doi:10.1007/s00127-026-03048-w)
Supplement: Supplementary file 1 — Supplementary Material 1 [file 127_2026_3048_MOESM1_ESM.docx]

**Supplementary 1: Data missing for total sample**

| **Variable** | **N missing** | **% Data missing** |
| --- | --- | --- |
| **Early childhood predictors** | | |
| Sex | 0 | 0% |
| House type | 28 | 8.9% |
| Household member count | 28 | 8.9% |
| Caregiver employment | 28 | 8.9% |
| Infant attachment | 62 | 19.7% |
| Maternal sensitivity | 58 | 18.4% |
| Maternal depression | 26 | 8.3% |
| **Current predictors** | | |
| House type | 25 | 8.0% |
| Household member count | 25 | 8.0% |
| Household monthly income | 26 | 8.3% |
| Food insecurity | 25 | 8.0% |
| Family support | 24 | 7.6% |
| Friend support | 24 | 7.6% |
| Self-esteem | 24 | 7.6% |
| Exposure to violence | 25 | 8.0% |
| Caregiver alcohol use | 26 | 8.3% |
| Caregiver stress | 28 | 8.9% |
