# Supplementary material for: Early childhood and early adolescent predictors of internalising symptoms in adolescents: findings from a longitudinal study in a high-risk South African environment
Source: Soc Psychiatry Psychiatr Epidemiol. 2026 Feb 11;61(7):1293–304. doi: 10.1007/s00127-026-03048-w (PMC13424707; doi:10.1007/s00127-026-03048-w)
Supplement: Supplementary file 2 — Supplementary Material 2 [file 127_2026_3048_MOESM2_ESM.docx]

**Supplementary 2: Correlation between early childhood and early adolescent predictors**

|  | **1** | **2** | **3** | **4** | **5** | **6** | **7** | **8** | **9** | **10** | **11** | **12** | **13** | **14** | **15** | **16** | **17** |
| --- | --- | --- | --- | --- | --- | --- | --- | --- | --- | --- | --- | --- | --- | --- | --- | --- | --- |
| **1.Sex** | 1 | -0.03 | 0.08 | -0.05 | -0.03 | -0.05 | -0.04 | 0.01 | 0.03 | 0.05 | -0.03 | -0.09 | -0.17** | 0.03 | 0.02 | -0.03 | 0.04 |
| **2.House type** | -0.03 | 1 | -0.03 | 0.07 | 0.03 | -0.05 | 0.05 | 0.17** | 0.05 | 0.06 | 0.04 | -0.05 | 0.05 | 0.06 | 0.04 | 0.06 | 0.01 |
| **3.Household members** | 0.08 | -0.03 | 1 | -0.02 | 0.04 | 0.06 | 0.02 | -0.003 | 0.16* | 0.04 | 0.03 | -0.001 | -0.001 | -0.04 | -0.01 | -0.03 | 0.02 |
| **4.Caregiver employment** | -0.05 | 0.07 | -0.02 | 1 | 0.06 | 0.09 | 0.001 | 0.06 | 0.07 | 0.01 | 0.02 | -0.05 | 0.12 | -0.01 | 0.02 | -0.08 | 0.02 |
| **5.Infant attachment** | -0.03 | 0.03 | 0.04 | 0.06 | 1 | 0.04 | -0.02 | 0.07 | -0.04 | 0.10 | -0.04 | 0.01 | -0.09 | -0.08 | -0.02 | -0.13* | 0.14* |
| **6.Maternal sensitivity** | -0.05 | -0.05 | 0.06 | 0.09 | 0.04 | 1 | -0.01 | 0.02 | 0.01 | 0.14* | 0.03 | -0.07 | -0.08 | -0.04 | 0.02 | -0.08 | 0.05 |
| **7.Maternal depression** | -0.04 | 0.05 | 0.02 | 0.001 | -0.02 | -0.01 | 1 | 0.02 | 0.07 | -0.04 | -0.02 | 0.03 | -0.11 | -0.04 | 0.12* | 0.21** | -0.22** |
| **8.House type** | 0.01 | 0.17** | -0.003 | 0.06 | 0.07 | 0.02 | 0.02 | 1 | 0.08 | 0.10 | -0.07 | -0.02 | 0.10 | 0.08 | -0.01 | -0.05 | 0.12* |
| **9.Household members** | 0.03 | 0.05 | 0.16* | 0.07 | -0.04 | 0.01 | 0.07 | 0.08 | 1 | 0.07 | 0.04 | -0.05 | -0.01 | 0.11 | 0.07 | -0.06 | -0.06 |
| **10.Household income** | 0.05 | 0.06 | 0.04 | 0.01 | 0.10 | 0.14* | -0.04 | 0.10 | 0.07 | 1 | -0.41** | -0.03 | 0.07 | -0.03 | -0.03 | -0.20* | 0.22* |
| **11.Food insecurity** | -0.03 | 0.04 | 0.03 | 0.02 | -0.04 | 0.03 | -0.02 | -0.07 | 0.04 | -0.41* | 1 | -0.06 | -0.06 | -0.04 | 0.09 | 0.14* | -0.23** |
| **12.Family support** | -0.09 | -0.05 | -0.001 | -0.05 | 0.01 | -0.07 | 0.03 | -0.02 | -0.05 | -0.03 | -0.06 | 1 | 0.11 | 0.22** | -0.05 | -0.03 | -0.07 |
| **13.Friend support** | -0.17** | 0.05 | -0.001 | 0.12 | -0.09 | -0.08 | -0.11 | 0.10 | -0.01 | 0.07 | -0.06 | 0.11 | 1 | 0.13* | -0.12 | 0.05 | 0.16** |
| **14.Self-esteem** | 0.03 | 0.06 | -0.04 | -0.01 | -0.08 | -0.04 | -0.04 | 0.08 | 0.11 | -0.03 | -0.04 | 0.22* | 0.13* | 1 | 0.02 | -0.04 | 0.15* |
| **15.Violence exposure** | 0.02 | 0.04 | -0.01 | 0.02 | -0.02 | 0.02 | 0.12* | -0.01 | 0.07 | -0.03 | 0.09 | -0.05 | -0.12 | 0.02 | 1 | 0.05 | -0.18** |
| **16.Caregiver alcohol use** | -0.03 | 0.06 | -0.03 | -0.08 | -0.13* | -0.08 | 0.21** | -0.05 | -0.06 | -0.20** | 0.14* | -0.03 | 0.05 | -0.04 | 0.05 | 1 | -0.21** |
| **17.Caregiver stress** | 0.04 | 0.01 | 0.02 | 0.02 | 0.14* | 0.05 | -0.22** | 0.12* | -0.06 | 0.22** | -0.23** | -0.07 | 0.16** | 0.15* | -0.18** | -0.21** | 1 |
| p < 0.05; **p < 0.01 | | | | | | | | | | | | | | | | | |
